# Parkinson’s Disease Mild Cognitive Impairment with MRI evidence of Cholinergic Nucleus 4 Degeneration: A New Subtype?

**DOI:** 10.21203/rs.3.rs-5278177/v1

**Published:** 2024-11-11

**Authors:** Ahmed Negida, Hiba Vohra, Sarah Lageman, Nitai Mukhopadhyay, Brian Berman, Daniel Weintraub, Matthew Barrett

**Affiliations:** Virginia Commonwealth University; Virginia Commonwealth University; Virginia Commonwealth University; Virginia Commonwealth University; Virginia Commonwealth University; University of Pennsylvania, Philadelphia Veterans Affairs Medical Center; Virginia Commonwealth University

**Keywords:** Parkinson’s Disease, Subtyping, Mild Cognitive Impairment, Cholinergic Nuclei

## Abstract

Subtyping Parkinson’s disease with mild cognitive impairment (PD-MCI) could improve clinical trial design and personalized treatments. Cholinergic nucleus 4 (Ch4) volume has been linked to cognitive impairment severity and future decline in PD. This study investigates whether PD-MCI patients with MRI evidence of Ch4 degeneration have distinct clinical profiles and cognitive trajectories. Baseline MRI scans of 148 PD-MCI participants from the Parkinson’s Progression Markers Initiative (PPMI) were analyzed. Patients with low Ch4 grey matter density (GMD) had worse motor, autonomic, and olfactory symptoms, and were more likely to belong to the diffuse malignant PD subtype (51.6% vs. 23.4%; P < 0.01). They also had faster progression to cognitive milestones (P = 0.0046). These findings identify PD-MCI with low Ch4 as a distinct subtype with more severe symptoms and faster cognitive decline, highlighting the importance of considering this group in PD-MCI clinical trials, particularly for cholinergic therapies.

## 1 Introduction

Mild cognitive impairment (MCI) is a common non-motor symptom of Parkinson’s Disease (PD) and is associated with worse quality of life and increased risk of developing dementia ^1^. There are currently no proven pharmacological therapies that improve or slow progression of PD with MCI (PD-MCI) ^2^. One of the main reasons why there are no proven effective symptomatic therapies for PD-MCI is because it is a heterogenous entity with multiple contributing pathologies. Indeed, PD-MCI has been associated with Lewy body pathology, Alzheimer’s disease (AD) co-pathology, and cerebrovascular disease, as well as cholinergic, and dopaminergic and noradrenergic neurotransmitter deficiencies ^3^. Given the multiple contributing underlying pathologies, subtyping PD-MCI patients *in vivo* could provide a possible solution for identifying pathologically and clinically homogenous patient groups that are likely to benefit from therapies targeting specific pathologies.

As per the Movement Disorders Society Task Force on the definition of PD, “*clinical subtypes should only be delineated if there are clear data that demonstrate consistent, large differences in prognosis, predicted disease manifestations, or treatment.*” ^4^. Several hypothesis-based and data-driven models for PD subtyping have been proposed, including tremor-dominant and PIGD subtypes and the brain-first and body-first subtype ^5–8^. However, many of these subtyping models lack subtype stability and external validation. A recent review of subtyping in PD found that most studies defining subtypes of PD rated poorly regarding the clinical importance of differences and the potential treatment implications ^9^. One recent data-driven subtyping approach that has been increasingly used in clinical research classifies patients into diffuse malignant, intermediate, and mild motor predominant ^10^.

There have also been efforts to subtype PD-MCI using hypothesis-driven subtyping approaches. These include amnestic vs. non-amnestic subtypes, multiple domains vs. single domain impairment, and the dual syndrome subtypes (posterior-cortical syndrome vs. the frontal syndrome) ^6,11–14^. While these subtypes have been shown to predict differences in prognosis and manifestations, there have been concerns raised about subtype stability, external validity, and whether these subtypes represent distinct entities or different stages of disease ^1,6,15,16^. Most importantly, none of these subtyping models have informed therapeutic development.

One novel and promising way to subtype PD-MCI is based on cholinergic nucleus 4 (Ch4) degeneration, one of the key underlying pathologies contributing to PD-MCI. Ch4, which comprises the majority of the nucleus basalis of Meynert (NBM), provides widespread cholinergic innervation to the cortex and amygdala, and it play an important role in attention, executive function, and working memory ^17–21^. Imaging and autopsy studies identified cholinergic degeneration in early stages of PD and even before the onset of dementia ^22–27^. Post-mortem and PET imaging studies identified cholinergic system degeneration within the Ch4 in PD and PDD patients ^28–30^, and several studies have linked cholinergic system degeneration to PD cognitive decline ^17,31–33^. Microstructural changes and volume loss in the NBM can be detected before patients develop MCI ^34^ and the degree of cholinergic basal forebrain degeneration was found to correlate with the severity of cognitive impairment ^35–37^.

Ch4 volume can be measured with MRI using probabilistic maps applied to voxel-based morphometry (VBM). Application of this imaging methodology found that Ch4 degeneration precedes and predicts the onset of cognitive impairment in PD patients ^31^. The extent of Ch4 atrophy correlates with the severity of cognitive impairment ^38^. To the best of our knowledge, no prior studies have attempted to subtype PD-MCI patients with MRI evidence of Ch4 degeneration. The objective of this study was to determine if PD-MCI patients with MRI evidence of Ch4 degeneration have a differentiating clinical profile and more rapid cognitive decline compared to PD-MCI patients without evidence of Ch4 degeneration. Unlike previous PD-MCI subtyping models, our approach aims to identify a PD-MCI subtype with treatment implications, i.e., to identify PD-MCI patients who may be more likely to benefit from therapies targeting the cholinergic systems.

## 2 Methods

### 2.1 Study Design

We conducted an observational cross-sectional case-control analysis using data from the Michael J. Fox Foundation’s Parkinson’s Progression Markers Initiative (PPMI). The PPMI is a prospective, longitudinal, observational, multicenter study, which aims to verify biomarkers of PD progression. The institutional review board at each PPMI site approved the study and participants provided informed consent prior to participation. An institutional ethical approval from VCU was exempt for this study because it is a secondary analysis of de-identified PPMI data. We followed the standard reporting guidelines for observational studies (STROBE statement) for this study.

### 2.2 Study Participants

PD participants in the original PPMI study were 30 years or older and met the following criteria at their baseline visit: (1) had been diagnosed within the last 2 years, (2) would not need to begin any medications for PD in the next 6 months, (3) had at least 2 listed symptoms (resting tremor, bradykinesia, or rigidity), (4) with a Hoehn and Yahr State I or II, and (5) had a positive DaT scan (dopamine transporter deficit). PD participants also completed clinical and biomarker assessments every 6 months after baseline. Included data represent eligible participants from all records available when accessed on May 9th, 2023.

Details regarding PPMI methodology are available online (https://www.ppmi-info.org/study-design/). In this study, we only included PD-MCI participants who had completed brain T1-weighted (T1w) MRIs and neuropsychological assessments at the baseline visit. Participants with Montreal Cognitive Assessment (MoCA) score < 21 were excluded to reduce risk of including demented participants. PD participants were defined as MCI if they had )1) a MOCA score between 21 and 26 or )2) two or more neurocognitive test scores > 1.5 standard deviations below the mean. There were 24 participants for whom only the MoCA was available for cognitive categorization. According to PPMI methods, healthy controls had no significant neurologic dysfunction, no first-degree family member with PD, and a MoCA score ≥ 27. Out of the 181 healthy controls with T1w-MRI at baseline, we excluded 10 participants whose cognitive status was determined to be MCI by site investigators. Those 171 healthy controls had a mean age of 60.6±11.6 years and 105 (61.40%) of them were males.

### 2.3 Clinical Assessments and Outcomes

As part of the PPMI study, participants completed the following assessments: 1) MoCA, 2) Hopkins Verbal Learning Test - Revised, 3) Letter Number Sequencing, 4) Semantic Fluency Test, 5) Symbol Digit Modalities Test, 6) Benton Judgement of Line orientation, 7) Movement Disorders Society Unified Parkinson’s Disease Rating Scale (MDS-UPDRS), 8) University of Pennsylvania Smell ID Test (UPSIT), 9) Scales for Outcomes in Parkinson’s Disease-Autonomic questionnaire (SCOPA-AUT), and 10) REM Behavior disorder severity questionnaire (RBDSQ). Using MDS-UPDRS, SCOPA-AUT, RBDSQ, and MoCA, we applied the criteria developed by Fereshtehnejad *et al*.^10^ to determine each participant’s data-driven subtype. First, a composite motor score was calculated from the sum of MDS-UPDRS-II and MDS-UPDRS-III. For the non-motor scores, (1) SCOPA-AUT, (2) RBDSQ, and (3) MoCA, the 75th percentiles of these composite motor and non-motor scores were first calculated from the whole dataset of PD participants in the PPMI (n = 927). Then, diffuse malignant subtype was defined as patients with > 75th percentile in the composite motor score and at least one non-motor score or > 75th percentile in all 3 non-motor scores. Mild-motor predominant subtype was defined as patients with < 75th percentile in the composite motor score and all the 3 non-motor scores. Intermediate subtype was defined as patients who did not fit either of the 2 other subtypes. The percentiles used to define these data-driven subtypes were disease duration specific. In this way, we assigned the data-driven subtype that corresponded to the duration of disease at the time of MRI.

### 2.4 Cognitive Milestones

Out of the 25 disease progression milestones previously identified in the PPMI ^39^, 6 milestones are categorized in the cognitive domain as follows: Cognitive impairment defined as MoCA < 21, Cognitive impairment in the MDS-UPDRS item 1.1 defined as response ≥ 3, hallucinations defined as MDS-UPDRS item 1.2 response ≥ 3, apathy defined as MDS-UPDRS item 1.5 response ≥ 3, dementia (clinical diagnosis) by site investigator assessment, and dementia (composite categorization) using impairment on ≥ 2 cognitive domains with functional impairment assessed per investigator. We assessed whether the study participants reached any of these 6 cognitive milestones at any time point.

### 2.5 MRI Acquisition, Preprocessing, and Analysis

Depending on the PPMI site, baseline T1w-MRI scans were performed on Siemens, Philips and GE scanners. Regional grey matter density (GMD) was calculated for all subjects according to our previously published methods ^40–43^. First, all DICOM files downloaded from the PPMI were converted to NIfTI format. Patient ID and scan date were extracted from the image metadata. All MRIs were preprocessed using voxel-based morphometry pipeline. First, T1w MRIs were normalized to the standard Montreal Neurological Institute (MNI) template using Diffeomorphic Anatomical Registration Through Exponentiated Lie Algebra (DARTEL) to a 1.5-mm isotropic adult template ^44^. Then, normalized images underwent segmentation into grey matter, white matter, and cerebrospinal fluid using the Adaptive Maximum A Posteriori (AMAP) method ^45^. We used the tissue probability maps of Lorio *et al*.^46^ for spatial normalization, skull-stripping, and generating the initial segmentation estimates within the AMAP framework. Partial Volume Estimation was utilized to estimate fractions of voxels containing multiple tissue types ^47,48^. To register the segmented images with the MNI space, the DARTEL algorithm and Geodesic Shooting method were employed. Modulation was applied to the segmented images to account for volume changes during spatial registration, ensuring that the total grey matter volume was preserved. For quality assurance, the resulting images were inspected visually. Finally, GM images were smoothed using an 8-mm full width at half maximum Gaussian kernel. The above procedures were done using SPM12 and the computational anatomy toolbox (CAT12, https://neuro-jena.github.io/cat/), which is a powerful toolbox to examine the structural alterations in brain grey matter volumes ^49^. Total intracranial volumes (TIV) were estimated from CAT12 during the segmentation module. Ch4 GMD was measured using MNI-normalized probabilistic maps that were derived from 3D reconstruction of histological sections from 10 post-mortem human brains ^50^. We calculated the relative GMD by multiplying the sum of grey matter for each voxel by the weighting contained within the probabilistic map as previously reported ^51^. This relative GMD was standardized by dividing by the total weighting in the mask.

### 2.6 Norming of Ch4 Grey Matter Density

Using T1w-MRIs of 171 healthy controls, we normed PD-MCI Ch4 GMD based on age, sex, TIV, and scanner type. Ch4 GMD values for healthy controls were converted to scaled scores (mean 10, SD 3). Next, the resulting scaled scores were regressed on the 4 variables. To obtain adjusted Ch4 Z-scores for the PD-MCI patients, we first converted each participant’s Ch4 GMD value to a scaled score based on the healthy controls’ values. Predicted scores were calculated using the β-coefficients for all 4 variables and their predictive constant using the following formula:

$$Scaled\;score_{predicted}=constant+b_{age}\left(age\right)+b_{sex}\left(sex\right)+b_{GE}\left(GE\right)+b_{phillips}\left(phillips\right)+b_{TIV}\left(TIV\right)$$

Then, the predicted scaled scores were subtracted from each participant’s actual scaled score and the difference was divided by the root mean squared error (RMSE) of the HCs: *z*-score = (scaled score_actual_ - scaled score_predicted_)/RMSE. PD-MCI participants were classified as low Ch4 (Z ≤−1 SD) or normal Ch4 (Z >−1 SD) based on the z-scores of their Ch4 GMD derived from this regression-based norming method.

### 2.7 Study Size and Power Calculation

The sample size of 148 PD patients (n = 116 vs. 32) who were found to be eligible for this study, provided 80% statistical power to demonstrate a moderate effect size (standardized mean difference of 0.56) between the two study groups in any clinical measure with a 5% two-tailed margin of error. This power calculation was performed using the jpower modules of Jamovi (version 2.3 for macOS) ^52^.

### 2.8 Statistical Analysis

Data were described as median and interquartile ranges for continuous variables or percentages and ratios for categorical variables. Comparisons between the two study groups were done using the Kruskal-Wallis test for continuous variables and the Pearson chi-square for categorical variables. Time-to-cognitive milestones was assessed using the Kaplan-Meier survival analysis method. We used the log-rank test to compare the time to reach cognitive milestones between groups and the multivariable cox-regression model to test the association between low Ch4 GMD and time to reach cognitive milestones while adjusting for age, sex, and MoCA. For all statistical tests, a p-value < 0.05 was considered statistically significant. Statistical analysis was performed using StataMP (version 17 for macOS).

## 3 Results

### 3.1 Clinical Characteristics of the Study Groups

T1w MRI scans were available for 148 PD-MCI participants from the PPMI database. According to their Ch4 GMD regressed Z score, 116 were identified as having normal Ch4 GMD and 32 were identified as having low Ch4. Clinical characteristics of the study groups are shown in Table 1. Regarding age and sex, there were no statistically significant differences between the two groups. PD-MCI patients with low Ch4 GMD had worse UPSIT, SCOPA-AUT, MDS-UPDRS part 1, MDS-UPDRS part II, MDS-UPDRS part III, and total MDS-UPDRS scores compared to those with normal Ch4 GMD.

### 3.2 MDS-UPDRS Part 1 subscales in the Study Groups

For the MDS-UPDRS part 1 subscales, PD-MCI patients with low Ch4 GMD had significantly more hallucinations and apathy compared to PD-MCI patients with normal Ch4 GMD (Table 2). Cognitive impairment, depressed mood, anxiety, and dopamine dysregulation syndrome were more frequent in the low Ch4 GMD group, but the differences did not reach statistical significance (P > 0.05).

### 3.3 Data-driven subtypes in Ch4 GMD groups

**Figure 2:**
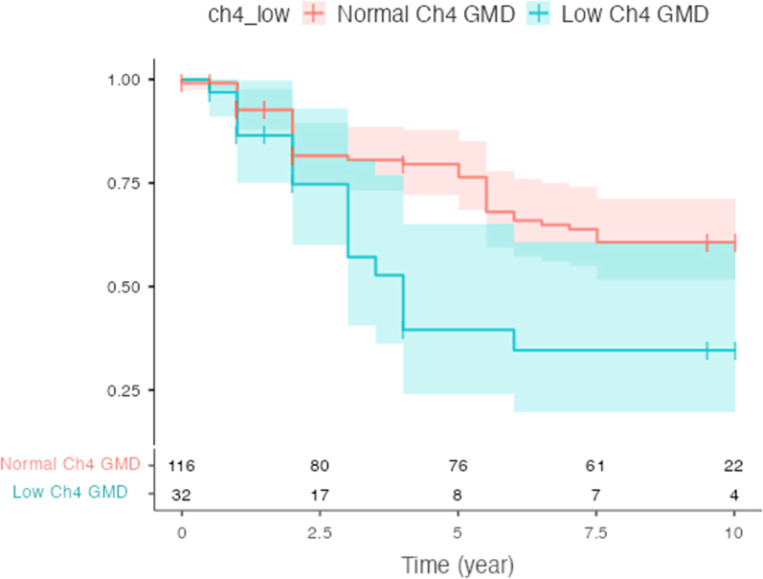
Kaplan-Meier Survival curve for the time-to-cognitive progression milestones

The frequency of the PD data-driven subtypes was significantly different between the study groups (Table 3). PD-MCI participants in the low Ch4 GMD group were two time more likely to meet criteria for diffuse malignant PD compared to the normal Ch4 group. Further, none of the participants in the low Ch4 GMD had mild-motor predominant PD (Fig. 2).

### 3.4 Time to reaching cognitive progression milestones

**Figure 3:**
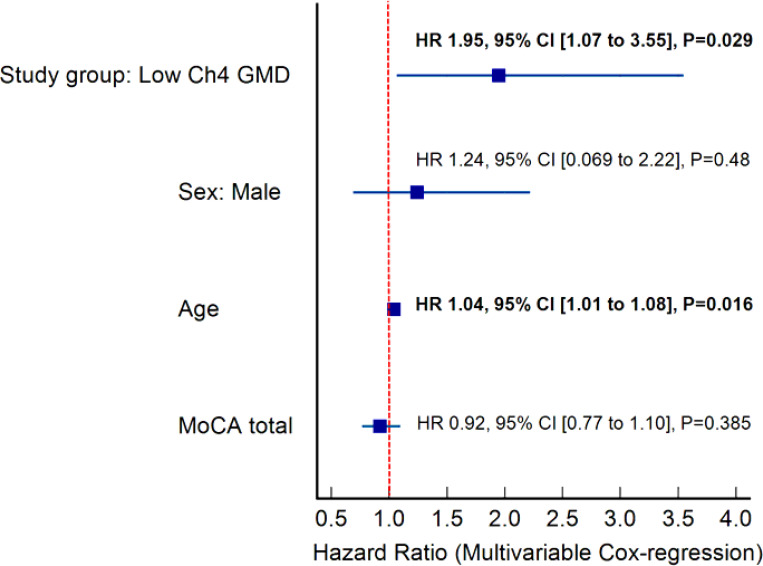
Forest plot for the HR and 95% confidence interval of the association between low Ch4 GMD and time-to-reach cognitive domain milestones, adjusted for age, sex, and MoCA.

The time to reaching any of the cognitive progression milestones was reduced in the low Ch4 GMD group compared to those with normal Ch4 GMD (log-rank *P* = 0.0046, Fig. 3), consistent with cognitive deterioration being faster in PD-MCI with low Ch4 GMD.

We performed a multivariable Cox regression analysis to assess the association between low Ch4 GMD and time-to-reach cognitive progression milestones. The analysis adjusted for sex, age, and total MoCA scores. The results are summarized in the forest plot (Fig. 4). Low Ch4 GMD was a significant predictor of earlier cognitive progression; patients with low Ch4 GMD exhibited a 1.95-fold increased hazard of reaching cognitive milestones compared to those with normal Ch4 GMD (HR = 1.95, 95% CI: 1.07–3.55, P = 0.029).

## 4 Discussion

In this study we found that PD-MCI participants with low Ch4 GMD had significantly worse MDS-UPDRS total score and its subscales (part I, II, and III) compared to PD-MCI with normal Ch4 GMD. PD-MCI with low Ch4 had more olfactory impairment, autonomic symptoms, psychotic symptoms, and apathy, and they were more likely to meet criteria for the diffuse malignant subtype of PD. Not only was their clinical profile distinct but PD-MCI participants with low Ch4 had a faster time to reach the cognitive progression milestones. These findings indicate that PD-MCI with low Ch4 GMD is associated with a more severe clinical profile and has a faster cognitive decline trajectory.

Our findings are consistent with a large body of work showing that reduced Ch4 GMD is associated with greater impairment in a range of motor and non-motor symptoms of PD. First, reduced Ch4 GMD in PD was found to be associated with cognitive impairment and greater risk of cognitive decline. Second, prior work also found that evidence of Ch4 degeneration was associated with psychotic symptoms ^42^, apathy 40, olfactory dysfunction ^41^, and autonomic dysfunction in PD ^42,53,54^. Third, two previous studies found that NBM grey matter volume and Ch4 GMD negatively correlated with the motor score (MDS-UPDRS-III) in PD patients ^55,56^, and numerous studies reported that greater gait impairment was linked to Ch4 degeneration ^56–61^. Further, a diffusion-weighted imaging study showed that patients with PIGD-PD had greater loss of microstructural integrity of the NBM white matter tracts compared to those with TD-PD ^59^. The novelty of our study is that we examined the fullest possible range of clinical correlates of Ch4 GMD in PD and examined these correlates in PD-MCI only. In doing so we found that PD-MCI participants with low Ch4 exhibit a more severe clinical profile across a variety of motor and non-motor symptoms.

We also sought to determine the overlap between the proposed subtype of PD-MCI with evidence of Ch4 degeneration and diffuse malignant PD, a data-driven subtype that was first proposed in 2015 ^62^ and validated in 2017 ^10^. Patients with diffuse malignant PD tend to have more prominent early motor and non-motor symptoms, faster disease progression, more symptoms of rapid eye movement sleep behavior disorder (RBD), mild cognitive impairment, orthostatic hypotension, and worse response to levodopa treatment ^10,63,64^. Interestingly, PD-MCI patients with low Ch4 were twice more likely to have the diffuse malignant subtype. In other words, within the PD-MCI population, reduced Ch4 volume is aligned with the more severe diffuse malignant subtype of PD, reinforcing the role of Ch4 degeneration in a more aggressive disease course.

The key clinical criteria for PD subtyping are biomarker profile and longitudinal progression ^5,10^ and the MDS definition of PD subtype implies that a new PD subtype should have specific prognostic implications in predicting the disease progression trajectory. To examine the disease trajectory PD-MCI with and without evidence of Ch4 degeneration, we used survival analysis to compare the time-to-reach cognitive progression milestones ^39^. We found that PD-MCI with low Ch4 had faster time to cognitive domain milestones (Fig. 3) ^38,65^. These results imply that this subtype has a prognostic value and clinical implications; since those patients are more likely to convert to dementia, they should be prioritized for clinical trials aiming to slow or prevent the cognitive deterioration in PD-MCI population. A possible explanation for why this distinct PD-MCI subtype has more rapid progression is that they lack compensatory upregulation in the cholinergic system which has previously been shown in PD with preserved cognition ^66^.

A limitation of this study is the cross-sectional of these data and the lack of information about the impact of longitudinal changes in Ch4 GMD on the disease course. Another requirement for reliable PD subtyping studies is subtype stability. Owing to the cross-sectional nature of this study and limited sample size, we could not examine subtype stability over time. Because this study examines PD patients with MRI imaging at baseline visit of the PPMI, we did not examine patients who switched from normal Ch4 GMD at baseline to low Ch4 GMD in subsequent follow-up visits. Future studies should examine the disease progression in PD-MCI patients with degenerated Ch4 compared to those with normal Ch4.

In conclusion, PD-MCI patients with low Ch4 GMD represent a distinct subtype marked by more severe motor and non-motor symptoms, rapid cognitive decline, and alignment with the diffuse malignant PD subtype. The association between evidence of Ch4 degeneration and faster progression to dementia highlights the urgency of prioritizing these patients for clinical trials. Given the evidence for cholinergic degeneration in PD-MCI with low Ch4, this PD-MCI subtype represents a population more likely to benefit from targeted cholinergic therapies, potentially improving both cognitive and non-cognitive symptoms. Future longitudinal studies should confirm the stability of this subtype and further explore therapeutic strategies to improve cognition or slow cognitive deterioration in this high-risk group.

## Figures and Tables

**Figure 1:**
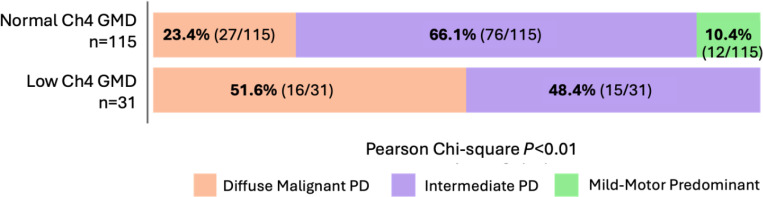
The distribution of PD data-driven subtypes in the study groups

**Table 1:** The baseline demographic and clinical characteristics of the PD-MCI study groups

	N	Normal Ch4 GMD (N = 116)	Low Ch4 GMD (N = 32)	P value
Age at enrollment	148	64.2 (56.7–69.7)	65.8 (60.6–70.8)	P = 0.18
Age at MRI	148	64.3 (57.0–69.7)	65.8 (60.6–70.9)	P = 0.17
Disease duration at MRI	148	0.5 (0.2–1.3)	0.5 (0.2–1.2)	P = 0.73
Sex: Male	148	65.5% (76/116)	75% (24/32)	P = 0.31
MDS-UPDRS Part 1	148	1.0 (0.0–2.0)	1.0 (0.4–4.0)	P = 0.04*
MDS-UPDRS Part 2	148	5.0 (2.0–8.0)	7.0 (3.4–11.6)	P = 0.01*
MDS-UPDRS Part 3 (off)	143	22.0 (17.0–29.6)	27.0 (21.0–32.7)	P = 0.04*
MDS-UPDRS Total (off)	143	32.0 (25.0–42.0)	38.0 (29.7–48.8)	P = 0.01*
UPSIT total	126	22.0 (14.2–28.0)	16.0 (10.3–20.7)	P < 0.01*
RBDSQ total	148	3.0 (2.0–5.0)	4.0 (2.0–8.0)	P = 0.13
SCOPA-AUT	148	11.5 (6.0–25.6)	19.5 (11.0–30.2)	P = 0.03*
MoCA score	148	25.0 (24.0–25.0)	24.0 (22.4–25.0)	P = 0.05
HVLT-R delayed recall	124	39.0 (32.0–45.0)	38.0 (32.0–46.3)	P = 0.98
HVLT-R total recall	124	41.5 (31.4–48.0)	39.5 (34.0–48.3)	P = 0.92
Semantic fluency, animals	124	48.0 (41.0–55.6)	47.0 (37.8–53.0)	P = 0.58
SDMT	124	42.5 (36.2–51.0)	39.8 (29.0–46.9)	P = 0.17
LNS	124	11.0 (9.0–12.0)	10.0 (8.0–12.0)	P = 0.33
JLO	124	11.7 (9.2–13.7)	10.4 (6.5–12.4)	P = 0.05

Continuous variables are reported as medians (interquartile range). N is the number of non-missing values. Cognitive scores are reported as T-scores unless otherwise stated. Abbreviations: HVLT-R = Hopkins Verbal Learning Test - revised; JLO = Judgment of Line Orientation; LNS = Letter Number Sequencing; MDS-UPDRS = Movement Disorder Society – Unified Parkinson’s Disease Rating Scale; MoCA = Montreal Cognitive Assessment; MRI = Magnetic Resonance Imaging; RBDSQ = REM Sleep Behavior Disorder Screening Questionnaire; SCOPA-AUT = Scales for outcomes in Parkinson’s disease - autonomic dysfunction; SDMT = Symbol Digit Modalities Test; UPSIT = University of Pennsylvania Smell Identification Test;

* Statistically significant (two-sided)

**Table 2:** Comparison between the study groups in terms of the MDS-UPDRS part 1 items

	Normal Ch4 GMD (N = 116)	Low Ch4 GMD (N = 32)	P value
Cognitive impairment	31.9% (37/116)	43.8% (14/32)	P = 0.21
Hallucinations	1.7% (2/116)	18.8% (6/32)	P < 0.01*
Depressed mood	25.9% (30/116)	37.5% (12/32)	P = 0.20
Anxiety	40.5% (47/116)	46.9% (15/32)	P = 0.52
Apathy	12.9% (15/116)	28.1% (9/32)	P = 0.04*
Dopamine dysregulation syndrome	0.0% (0/116)	3.1% (1/32)	P = 0.06

Participants were considered as having a symptom if they scored 1 or more on each item.

* Statistically significant (two-sided)

## Data Availability

The datasets analyzed during the current study are publicly available from the Michael J. Fox’s PPMI database: http://PPMI-info.org. The statistical analysis files: Stata script files (.do), Jamovi files (.omv), and the final dataset (.csv) are available from the corresponding author on reasonable request.
